# Role of m6A modification in regulating the PI3K/AKT signaling pathway in cancer

**DOI:** 10.1186/s12967-023-04651-0

**Published:** 2023-11-01

**Authors:** Jie Liu, Xinyu Gu, Zhenjie Guan, Di Huang, Huiwu Xing, Lian Zheng

**Affiliations:** 1 Department of Oral and Maxillofacial Surgery, The First Affiliated Hospital of Zhengzhou University, No. 1 Jianshe East Road, Erqi District, Zhengzhou, 450052 Henan China; 2 Department of Oncology, The First Affiliated Hospital, College of Clinical Medicine, Henan University of Science and Technology, Luoyang, 471000 Henan China; 3 Department of Stomatology, The First Affiliated Hospital of Zhengzhou University, Zhengzhou, 450052 Henan China; 4 Department of Child Health Care, The Third Affiliated Hospital of Zhengzhou University, Zhengzhou, 450000 Henan China; 5 Department of Pediatric Surgery, The First Affiliated Hospital of Zhengzhou University, No. 1 Jianshe East Road, Erqi District, Zhengzhou, 450052 Henan China

**Keywords:** M6A regulator, PI3K/AKT signaling pathway, Epithelial tumors, Prognosis

## Abstract

The phosphoinositide 3-kinase (PI3K)/AKT signaling pathway plays a crucial role in the pathogenesis of cancer. The dysregulation of this pathway has been linked to the development and initiation of various types of cancer. Recently, epigenetic modifications, particularly *N*6-methyladenosine (m6A), have been recognized as essential contributors to mRNA-related biological processes and translation. The abnormal expression of m6A modification enzymes has been associated with oncogenesis, tumor progression, and drug resistance. Here, we review the role of m6A modification in regulating the PI3K/AKT pathway in cancer and its implications in the development of novel strategies for cancer treatment.

## Introduction

Cancer is a complex and challenging disease that affects multiple physiological organs and systems [1, 2]. It is a significant contributor to morbidity and mortality worldwide [3, 4]. Extensive research has been conducted to understand the fundamental mechanisms and etiology of cancer, as well as develop effective diagnostic modalities and therapeutic interventions [1, 5]. Cancer treatment involves a multidisciplinary approach that includes surgical intervention, radiation therapy, anticancer drug treatment, targeted therapy, and immunotherapy [6–8]. Despite significant progress in cancer research and therapies, challenges still exist in the management of this disease [9, 10]. A comprehensive understanding of the fundamental mechanisms and determinants of cancer is necessary to comprehend its complexity [11].

The enzyme phosphatidylinositol 3-kinase (PI3K) catalyzes the conversion of phosphatidylinositol 4,5-bisphosphate to phosphatidylinositol-3,4,5-triphosphate. This secondary messenger stimulates the recruitment of AKT to the cell membrane. AKT is phosphorylated and becomes activated upon recruitment. AKT activation subsequently activates downstream targets involved in promoting cell growth and survival, such as the mammalian target of rapamycin (mTOR) [12–14]. The PI3K/AKT pathway, which regulates various aspects of cell growth, has been extensively studied due to its critical role in cancer development [15–17]. This pathway is commonly activated by genetic alterations (e.g., mutations or amplifications in PI3K or AKT genes) or abnormal activity of upstream signaling molecules (e.g., receptor tyrosine kinases) [18, 19]. The dysregulation of this pathway promotes oncogenic activities, such as increased cell proliferation, survival, and resistance to chemotherapy [20, 21].

Epigenetic changes, specifically RNA alterations through *N*6-methyladenosine (m6A) modification, have been recognized as universal intrinsic modifications of mRNA in several eukaryotic organisms, including mammals [22]. These modifications regulate the activity of the PI3K/AKT pathway [23–25]. The m6A modification involves adding a methyl moiety to the N6 position of adenosine residues within RNA molecules. This process is highly dynamic and reversible, and it modulates mRNA efficiency [24, 25]. The process is catalyzed by a set of enzymes, including m6A methyltransferases and demethylases, and proteins that recognize m6A-modified RNA [26].

The dysregulation of enzymes involved in m6A modification is associated with oncogenesis, tumor progression, and drug resistance [22, 24, 27, 28]. Aberrant expression levels of several m6A modification enzymes have been reported in multiple types of carcinomas [29–33]. These enzymes regulate the stability and translation of oncogenic mRNAs, including those involved in the PI3K/AKT signaling pathway, thereby promoting the activation of this pathway and the progression of cancer [34, 35].

## PI3K/AKT signaling in cancer

Phosphoinositide 3-kinases comprise a group of lipid kinases that are involved in regulating cellular processes, including cell cycle progression, apoptotic pathways, DNA damage response, and motility [36–38]. These enzymes are categorized into three distinct classes (Class I, II, and III) depending on the variations in their structure and function [39–41]. A hyperactive PI3K pathway is one of the most commonly observed phenomena in human cancers [42, 43]. This increased activity has been correlated with the onset of tumorigenesis, resistance to pharmacological interventions, and clinical prognosis [43]. The abnormal activation of the PI3K signaling pathway may be attributed to three main mechanisms [44], which are activating mutations or amplification of the catalytic subunits of PI3Ks, inactivation of the lipid phosphatase PTEN, and amplification or mutations of the receptors. Phosphatase and tensin homolog (PTEN) is a negative regulator of the PI3K/AKT pathway. Impaired PTEN function in somatic cells has been conclusively linked to an increased likelihood of prostate cancer metastasis and poorer prognoses [45–47].

The protein kinase AKT, also known as protein kinase B [48], was identified in the 1970s as an oncogene transduced by a transforming retrovirus called AKT-8. This retrovirus was isolated from a thymoma cell line derived from AKR mice. Later, in 1991, the AKT gene was cloned for the first time [49]. The AKT gene plays a central role in cellular signal transduction cascades that are activated by various growth factors, cytokines, and other cellular stimuli [50]. Abnormal activation of AKT plays a crucial role in the pathophysiological mechanisms underlying several complex diseases [51–54]. This process is controlled by various signaling pathways located upstream, such as the PI3K/Akt/mTOR pathway [52, 55]. The irregular functioning of the AKT pathway is commonly observed in various types of cancer and is associated with tumor growth, invasion, and metastasis [56–58]. Therefore, AKT is considered a potential target for cancer treatment, and numerous drugs are being developed to target AKT [59].

**Fig. 1:**
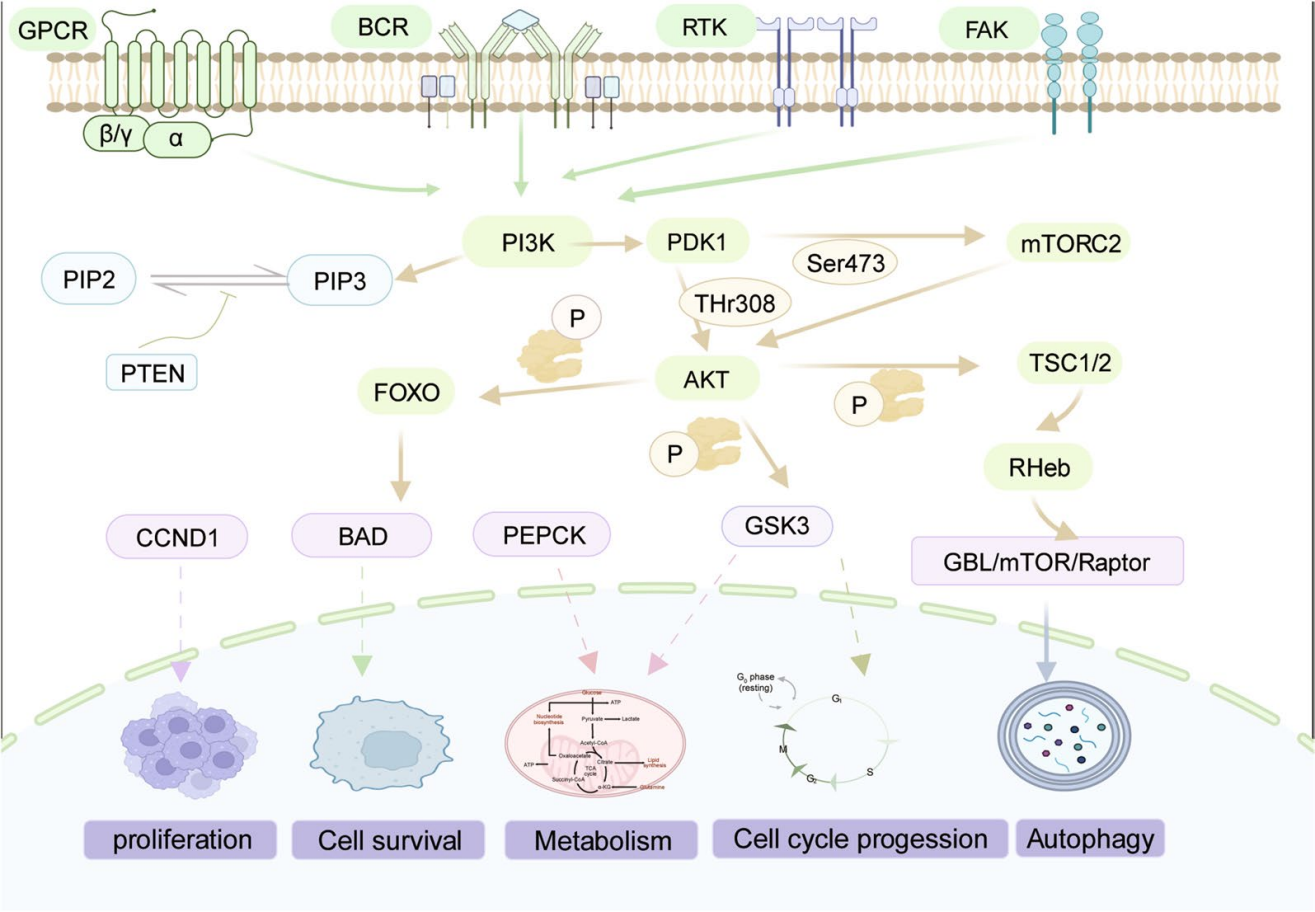
Overview of the PI3K/AKT signaling pathway. The PI3K/AKT signaling pathway is activated by GPCR, BCR, FAK, and RKT families. PIP3 triggers the activation of PDK1, which subsequently phosphorylates AKT at THr308. AKT modulates multiple intracellular biological processes by interacting with several downstream signaling molecules. The PI3K/AKT signaling pathway exerts a pivotal role in controlling diverse cellular processes, encompassing metabolism, growth, proliferation, survival, transcription, and protein synthesis. Image created with BioRender (https://biorender.com/)

The PI3K/AKT signaling pathway is involved in several cancer-associated processes [60–62], including cell survival, migration, and metabolic regulation [63]. In addition, this pathway actively participates in numerous key physiological processes that occur in the neoplastic microenvironment, such as inducing angiogenesis and recruiting inflammatory mediators [64]. The PI3K/AKT signaling pathway can be activated by multiple molecules, such as the RKT protein family, toll-like receptors, and B-cell receptors [64, 65]. The pathway is stimulated by the activation of receptor tyrosine kinases (RTKs) through the binding of various ligands, such as homologous growth factors, cytokines, and hormones [66, 67]. Among the RTKs, the 170-kDa epidermal growth factor receptors (EGFRs) are particularly important for activating the PI3K signal transduction pathway through tyrosine phosphorylation and the formation of homodimers/heterodimers in response to ligand binding [68–70]. The activation of the PI3K/Akt signaling pathway is influenced by B cell antigen receptor (BCR) and cytoplasmic adapters. Moreover, the absence of BCRs hinders the activation of AKT in B cells. These observations underscore the complex regulatory mechanisms that govern the PI3K signaling pathways and their involvement in cellular signaling events [71, 72] (Fig. 1).

## M6A regulators

*N*6-Methyladenosine modulates gene expression by regulating various cellular processes, such as cell self-renewal, differentiation, invasion, and apoptosis [73]. The regulators of m6A are divided into three distinct categories: writers, erasers, and readers [74–76]. “Writers” induce m6A modifications in the mRNA of tumor promoter or suppressor genes. “Readers” identify these modifications and subsequently increase or decrease the expression of tumor promoters or inhibitors, respectively. “Erasers” eliminate m6A and inhibit the activity of readers, thereby increasing the expression of tumor promoters or decreasing the expression of tumor suppressors [77–81]. Overall, RNA metabolism is regulated by these molecular interactions [82].

### Writers/m6A methyltransferases

The discovery of the methyltransferase-like 3 (METTL3) subunit as the sole catalytic protein in the methyltransferase complex was a significant milestone in this field [31]. METTL3 is a key member of a large and conserved family of putative SAM-dependent methyltransferases in mammals [83]. The METTL3 protein participates in neurogenesis and development by adding a methyl group at the N6 position (m6A) in the histone methyltransferase Ezh2, thereby enhancing its expression [84]. Choe et al. reported that the upregulated expression of METTL3 lead to an increase in the expression of ZMYM1 in gastric cancer cells. This increase accelerated the epithelial-to-mesenchymal transition (EMT) in vitro and facilitated metastasis in vivo [85].

Methyltransferase-like 5 (METTL5) catalyzes the methylation of RNAs, including U6 snRNA, 28S rRNA, and 18S rRNA. The enzyme shows autonomous catalytic activity and is not associated with the methyltransferase complex [86, 87]. METTL5 is an m6A methyltransferase that heterodimerizes with a coactivator, TRMT112. The binding stabilizes the enzyme and enhances its catalytic activity [88].

Methyltransferase-like 14 (METTL14) has a dual function as both tumor promoter and inhibitor in cancer pathogenesis [89–91]. The elimination of METTL14 increased the sensitivity of gastric cancer cells to cisplatin. This effect was achieved by stimulating apoptosis and autophagy through the mTOR signaling pathway, and suppressing the expression of Cytidine deaminase, consequently ameliorating the sensitivity of drug-tolerance cells towards gemcitabine (GEM) [92, 93].

Methyltransferase-like 16 (METTL16) has both methyltransferase activity-dependent and independent functions [94, 95]. In the cell nuclei, METTL16 acts as an m6A methyltransferase (“writer”) and introduces m6A modifications into specific mRNA targets. In the cytosol, it promotes the translation of numerous mRNA transcripts through an m6A-independent mechanism. METTL16 recruits eIF3a/b and rRNAs to facilitate the formation of the 43S pre-initiation complex and the 80S translation-initiation complex [95–98]. These dual roles of METTL16 contribute to its function in tumorigenesis [98–100].

Wilms’ tumor 1-associated protein (WTAP) is the subunit that recruits the m6A methyltransferase complex to specific mRNA targets. WTAP is essential for the accumulation of METTL3 and METTL14 in the nuclear space. Yunhao Chen et al. demonstrated that WTAP positively influences the m6A modification of ETS1 mRNA and inhibits the interaction between ETS1 mRNA and HuR [101].

The RBM15 gene is a member of the SPEN family, which is located on chromosome 1p13.3. The gene synthesizes the RBM15 protein, which is analogous to RBM15B [24, 102]. RBM15 and RBM15B lack enzymatic activity; however, they can physically associate with METTL3 and WTAP, thereby facilitating their localization to specific RNA sites for m6A modification [103, 104].

Zinc finger protein 217 (ZFP217) possesses a conserved zinc finger structure and is highly expressed in multiple types of cancer. The levels of ZFP217 are correlated with the prognosis of patients with cancer [105]. In contrast, ZC3H13 can act as a tumor suppressor. It plays a critical role in inhibiting the progression and metastasis of colorectal and breast cancers. This is achieved by regulating specific signaling pathways, such as the Ras/ERK pathway in colorectal cancer and the Wnt pathway in breast cancer [106–108].

### Readers

The YTH *N*6-methyladenosine RNA-binding protein (YTHDF) family consists of the m6A receptors. The cytoplasmic YTHDF family members include YTHDF1, YTHDF2, and YTHDF3 [26, 109]. YTHDF1 enhances mRNA translation, whereas YTHDF2 accelerates mRNA turnover. YTHDF3 has a dual regulatory function because it simultaneously promotes both mRNA translation and degradation pathways [110].

The insulin-like growth factor 2 mRNA-binding protein (IGF2BP) family consists of three distinct m6A readers: IGF2BP1, IGF2BP2, and IGF2BP3. IGF2BP1 and IGF2BP3 are carcinoembryonic proteins synthesized by malignant and embryonic tissues [111, 112]. However, their levels decline in adult tissues. These proteins increase mRNA stability by interacting with target transcripts [113].

HnRNPA2/B1 directly binds to m6A-modified transcripts and is involved in regulating the processing of modified transcripts. A specific group of primary miRNA transcripts is processed by interacting with the miRNA microprocessor complex protein DGCR8 [114, 115].

### Erasers/demethylases

ALKBH5 has been identified as a mammalian m6A RNA demethylase, displaying demethylation activity both in vitro and in vivo. Its role in mRNA export, along with its association with nuclear speckle proteins and RNA metabolism, highlights the significance of ALKBH5 and its demethylation activity in these processes [116]. The FTO enzyme exhibits formidable demethylase activity directed towards a variety of RNA substrates that are methylated. This activity influences the splicing, stability, decay, and translation of messenger RNA. The primary location of FTO is found within the nucleus and cytoplasm [117]. FTO stimulates the development and progression of liver carcinoma, lung cancer [118], and breast cancer [119]. Nevertheless, FTO may have a suppressive effect on tumor growth in kidney [120], pancreatic [121], and thyroid cancers [122]. ALKBH5, discovered after FTO, is a crucial m6A demethylase involved in various types of cancer [121, 123, 124].

## Biological functions and mode of action of m6A regulators in multiple cancers

**Fig. 2:**
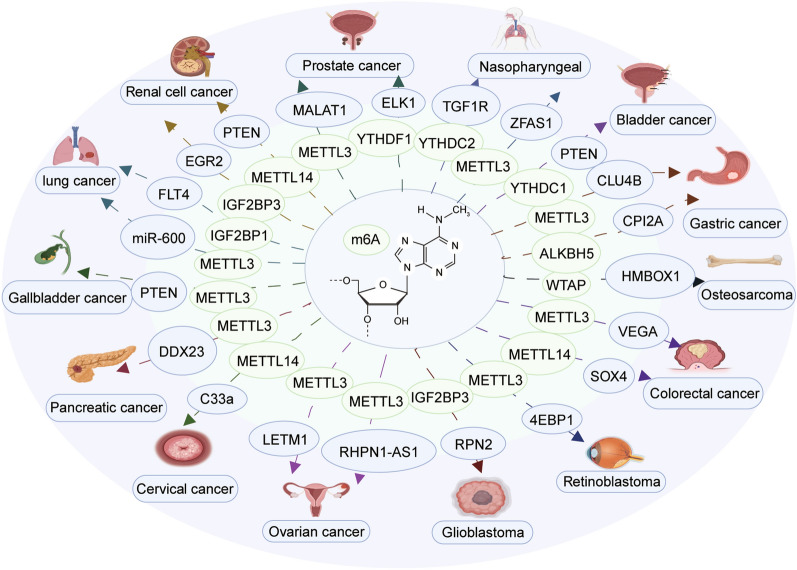
Interactions among the PI3K/AKT signaling pathway and m6A regulators in different types of cancers. M6A regulators interact with the PI3K/AKT signal transduction pathway, which critically regulates the biological functions in multiple types of tumors, including tumors of the digestive, respiratory, reproductive, urinary, and nervous systems. Image created with BioRender (https://biorender.com/)

The PI3K-AKT pathway is one of the most frequently activated pathways in human malignancies [63]. The PI3K/AKT pathway plays a crucial role in regulating various cellular processes, including metabolism, growth, proliferation, survival, transcription, and protein synthesis. Therefore, the PI3K-AKT pathway is a critical signaling cascade in human cancers [125, 126]. Several authors have suggested that a m6A modification influences the expression of key genes involved in the PI3K/AKT signaling cascade. The m6A/PI3K/AKT signaling cascade participates in the pathogenesis and progression of several neoplasms (Fig. 2). This section elaborates on the association between m6A regulators and clinical prognosis in various types of cancer (Table 1). Moreover, the functions of m6A regulators in relation to the PI3K/AKT signaling pathway in cancer have also been summarized in Table 2.

**Table 1:** Correlation between m6A regulators and clinical prognosis

Cancer type	Number of clinical samples	Regulator	Role in PI3K/AKT pathway	Role in cancer	Associated clinical features	Prognosis	References
Pancreatic cancer	–	METTL3	Activation	Oncogene	–	Poor overall survival	[130]
	32 adjacent normal pancreatic tissues and 32 pancreatic cancer tissues	METTL3/METTL14	Activation	Oncogene	Higher tumor stages	Poor overall survival	[131]
Gastric cancer	82 matched normal and cancer tissues	ALKBH5	Inhibition	Tumor suppressor	TNM categories and lymph node metastasis stage	Good overall survival	[136]
	72 fresh gastric cancer tissues	METTL3	Activation	Oncogene	Lymph node metastasis	Poor overall survival	[35]
	248 cancer tissue samples and matched adjacent tissue samples	METTL14	Inhibition	Tumor suppressor	TNM stage	Good overall survival	[137]
	Tissue specimens from 78 patients with diffuse gastric cancer	METTL14	Inhibition	Tumor suppressor	Worse clinical outlook (progressed TNM status and advanced tumor stages)	Good overall survival	[23]
Gallbladder cancer	Surgically removed 243 gallbladder cancer and paired non-tumor tissue samples	METTL3	Activation	Oncogene	–	Poor overall survival	[198]
Colorectal cancer	8 colorectal cancer and paired adjacent normal tissue specimens	METTL3	Activation	Oncogene	–	Poor overall survival	[142]
	136 colorectal cancer and corresponding adjacent normal tissues	METTL14	Inhibition	Tumor suppressor	Lymph node metastasis, distant metastasis and TNM stage	Good overall survival	[143]
Hepatocellular carcinoma	108 pairs of hepatocellular carcinoma and adjacent normal tissues	ALKBH5	Inhibition	Tumor suppressor	–	Good overall survival	[151]
	40 samples	METTL14	Inhibition	Tumor suppressor	Advanced TNM stage, and distant metastasis	Relapse-free, progression-free, and disease-specific survival	[152]
	26 tumor and adjacent matched non-tumor tissue samples	ALKBH5	Inhibition	Oncogene	Tumor size, tumor stage, TNM stage, and microvascular invasion	Lower overall survival	[153]
	117 pairs of primary hepatocellular carcinoma and their adjacent normal specimens	FTO	Activation	Oncogene	Metastatic nodule	Poor overall survival	[154]
	10 paired samples were analyzed using RNA-seq and 35 paired samples were analyzed using QRT-PCR and western blot analysis	IGF2BP1	Activation	Oncogene	Distant metastases	Poor overall survival	[155]
	12 tumor and matched adjacent nontumor liver tissue specimens	YTHDF1	Activation	Oncogene	Tumor weight and volume	Poor overall survival	[34]
Ovarian cancer	Ovarian cancer and corresponding adjacent nontumor tissues were obtained from 50 patients	METTL3/RHPN1-AS1	Activation	Oncogene	Tumor weight and volume	Poor overall survival	[159]
	86 fresh frozen cancerous and paracancerous ovarian tissue samples	METTL3	Activation	Oncogene	Distant metastasis and death	Worse overall and disease-free survival	[160]
Osteosarcoma	40 metastatic and 64 non-metastatic osteosarcoma samples	WTAP	Inhibition	Oncogene	Tumor size, metastasis, and TNM stage	Poor overall survival	[195]
Renal cell cancer	24 paired kidney cancer tissues and adjacent non-cancerous tissues	IGF2BP	Activation	Oncogene	Clinical stage, nodal involvement, and distant metastasis	Poor overall survival	[168]
	28 pairs of renal cell cancer tissues and the corresponding adjacent non-cancer tissues	METTL14	Activation	Tumor suppressor	–	Good overall survival	[167]
Prostate cancer	484 non-paired prostate cancer tissue samples and 52 pair-matched samples	METTL3	Activation	Oncogene	Lymph node metastasis	Poor overall survival	[175]
	100 fresh frozen samples of prostate tumor and adjacent normal tissues	YTHDF1	Activation	Oncogene	High pathologic T and N stages	Poor overall survival	[176]
Glioblastoma	–	IGF2BP3	Activation	Oncogene	–	Poor overall survival	[179]
Retinoblastoma	–	METTL3	Inhibition	Oncogene	–	Poor overall survival	[184]
Nasopharyngeal carcinoma	Tumor tissues and matched adjacent tissues from 53 patients with nasopharyngeal carcinoma	METTL3	Inhibition	Oncogene	Late stage and lymph node metastasis	Poor overall and disease-free survival	[188]
	105 paraffin-embedded primary nasopharyngeal carcinoma samples	YTHDC2	Activation	Oncogene	–	Poor overall survival	[189]
Lung cancer	–	METTL3	Activation	Oncogene	–	Poor overall survival	[192]
	2 lung cancer tissue samples with high CD34 expression and 2 with low CD34 expression	IGF2BP2	Activation	Oncogene	–	Poor overall survival	[193]

**Table 2:** Functions of m6A regulators related to the PI3K/AKT signaling pathway in cancer

Cancer type	Model	Regulator	Expression of regulator	Targets	Function	Mechanism	References
Pancreatic cancer	In vitro/in vivo	METTL3	Up	DDX23	Promote gemcitabine resistance	Modifying DDX23 mRNA m6A methylation through PI3K/AKT signaling activation	[130]
	In vitro/in vivo	METTL3/METTL14	Up	PTEN	Promote cell proliferation, EMT and tumorigenesis in PC cells	MiR-380-3p activated the AKT pathway in cancer cells by degrading PTEN	[131]
Gastric cancer	In vitro/in vivo	ALKBH5	Down	–	Promote apoptosis of gastric cancer cells	Attenuating the PI3K/AKT pathway	[136]
	In vitro/in vivo	METTL3	Up	CUL4B	Promote proliferation and invasiveness	Transcriptionally repressing miR-22-3p and miR-320a	[35]
	In vitro	METTL14	Down	CDH1	Promote proliferation and invasiveness	Reduce RNA m6A methylation activated oncogenic Wnt/PI3K/AKT signaling	[23]
Gallbladder cancer	In vitro/in vivo	METTL3	Up	PTEN	Promote proliferation and invasiveness	DCA-induced downregulation of miR-92b-3p inhibited oncogenic PI3K/AKT signaling by elevating PTEN	[198]
Colorectal cancer	In vitro/in vivo	METTL3	Up	EphA2/VEGFA	Promote proliferation and invasiveness	METTL3 methylated EphA2 and VEGFA induces VM formation by activating both PI3K/AKT and ERK1/2 signaling	[142]
	In vitro/in vivo	METTL14	Down	SOX4	Inhibits tumor metastasis	Promote SOX4-mediated EMT and activate SOX4-mediated PI3K/Akt signaling pathway	[143]
Hepatocellular carcinoma	In vitro	ALKBH5	Down	PAQR4	Suppress cancer cell proliferation, migration, and invasion	Downregulate PAQR4 expression in an m6A-dependent manner, suppress PI3K/AKT pathway activation	[151]
	In vitro/in vivo	METTL14	Down	EGFR	Promote proliferation and invasiveness	Inhibit cancer cell migration, invasion and EMT by modulating the EGFR/PI3K/AKT signaling pathway in an m6A-dependent manner	[152]
	In vitro/in vivo	ALKBH5	Down	SHIP2	Promotes cancer cell proliferation and metastasis, thereby conferring drug chemoresistance.	DestabilizedSHIP2 by enhancing E3 ubiquitin ligase CUL4A ubiquitination-dependent SHIP2 degradation	[153]
	In vitro/in vivo	FTO	Up	TGF-β2	Poor differentiation, and metastasis in patients with clinical hepatocellular carcinoma	RALYL could regulate HCC stemness through STAT3-dependent TGF-β2 upregulation	[154]
	–	IGF2BP1	Up	ATG16L1	Promote cell proliferation, migration and invasion	Adsorption of miR-346 and miR-874-3p to activate the PI3K/AKT/mTOR signaling pathway	[155]
	–	YTHDF1	Up	–	Promote the migration and invasion	Activate PI3K/AKT/mTOR signaling pathway and inducing EMT	[34]
Ovarian cancer	In vitro/in vivo	METTL3/RHPN1-AS1	Up	–	Promote cell viability, migration, invasion and tumor growth	METTL3-mediated m6A modification of RHPN1/AS1 accelerates cisplatin resistance in ovarian cancer by activating PI3K/AKT pathway	[159]
	In vitro/in vivo	METTL3	Up	LETM1	Promote cancer cell proliferation and metastasis	Sponge miR-596, increase LETM1 expression, and activate the FAK/PI3K/AKT signaling pathway	[160]
Cervical cancer	In vitro	METTL14	Down	–	Inhibit the growth and invasion of cervical cancer	Suppress the PI3K/AKT/mTOR signaling pathway by decreasing the phosphorylation of Akt and mTOR	[163]
Osteosarcoma	In vitro/in vivo	WTAP	Up	HMBOX1	Promote proliferation and invasiveness	WTAP/HMBOX1 regulate osteosarcoma growth and metastasis by regulating the PI3K/AKT pathway	[195]
Renal cell cancer	In vitro/in vivo	IGF2BP	Up	EGR2	Promote proliferation and invasiveness	Promote kidney tumorigenesis by activating the PI3K/AKT pathway	[168]
	In vitro/in vivo	METTL14	Down	PTEN	Promote the cell proliferation, migration and tumor progression	Restrain PTEN expression in clear cell renal cell carcinoma, leading to the tumor progression by activating the PI3K/AKT signaling pathway	[167]
Bladder cancer	In vitro/in vivo	YTHDC1	Down	PTEN	Promote proliferation and invasiveness	Lower YTHDC1 destabilizes PTEN mRNA, activates AKT-associated DNA damage response, and attenuates cisplatin-induced DNA damage	[171]
Prostate cancer	In vitro/in vivo	METTL3	Up	MALAT1	Promote proliferation and invasion	MALAT1 promotes the activation of PI3K/AKT signaling and abrogates METTL3 knockdown-induced PI3K/AKT signaling inactivation in prostate cancer cells	[175]
	In vitro/in vivo	YTHDF1	Up	PLK1	Promote proliferation, migration, and invasion	YTHDF1 regulates the PI3K/AKT signaling pathway through PLK1	[176]
Glioblastoma	In vitro/in vivo	IGF2BP3	Up	RPN2	Blocking WEE2-AS1 expression improved the therapeutic sensitivity to dasatinib	WEE2-AS1 promotes RPN2 protein stabilization by preventing CUL2-mediated RPN2 K322 ubiquitination	[179]
Retinoblastoma	In vitro/in vivo	METTL3	Up	–	Promote cell proliferation, migration and invasion of retinoblastoma cells	The PI3K/AKT/mTOR pathway regulates the translation of mRNAs that encode pro-oncogenic proteins, leading to malignant cell survival	[184]
Nasopharyngeal carcinoma	In vitro/in vivo	METTL3	Up	MiR-10-100p	Promote proliferation and invasiveness	ZFAS1 can regulate the expression of ATG10 through sponge miR-100-3p to affect the level of autophagy	[188]
	In vitro/in vivo	YTHDC2	Up	IGF1R	Promote radiotherapy resistance of nasopharyngeal carcinoma cells	Activate the PI3K/AKT/S6 pathway by regulating the translation of IGF1R mRNA	[189]
Lung cancer	In vitro	METTL3	Down	A549/H1299	Inhibit proliferation and migration	Induce apoptosis by inhibiting activation of the PI3K signaling pathway	[192]
	In vitro	IGF2BP2	Up	–	Promote angiogenesis and metastasis	Improve the RNA stability of FLT4 through m6A modification, thereby activating the PI3K/Akt signaling pathway	[193]

## Digestive system neoplasms

### Pancreatic cancer (PC) and pancreatic ductal adenocarcinoma (PDAC)

Pancreatic cancer is a highly fatal malignancy in humans, characterized by a high mortality rate. It is often diagnosed at advanced stages, and effective chemotherapy treatments have not been discovered to date. The majority of pancreatic tumors originate from the ductal epithelium, leading to the development of PDAC [127, 128]. The occurrence of pancreatic cancer is increasing annually at a rate of 0.5–1.0%. Based on projections, it is anticipated that pancreatic cancer will rise to become the second most common cause of cancer-related death in the United States by the year 2030 [129]. The expression levels of METTL3 and METTL14 are markedly increased in patients with PC [130, 131]. Lin et al. reported a positive correlation between elevated DEAD-box helicase 23 (DDX23) mRNA expression and the initiation of the PI3K/AKT signaling pathway, whereas, a negative correlation was observed between DDX23 and survival outcomes [130]. A synergistic reduction in the levels of miR-380-3p in PC cells when both METTL3 and METTL14 were deleted. Furthermore, patients with PC who have elevated miR-380-3p expression showed a poorer prognosis and higher tumor stages compared to those with reduced miR-380-3p expression [131]. Additionally, METTL3 enhanced GEM resistance and induced abnormal metabolism in PDAC cells [130]. MiR-380-3p appears to promote cell division, migration, and EMT in PC by regulating activation of the PTEN/AKT pathway [131]. METTL3 was elevated in GEM-resistant PC cells, and its downregulation inhibited cancer progression. Mechanistically, METTL3 promotes PDAC progression and gemcitabine resistance by modulating DDX23 mRNA m6A methylation and facilitating the initiation of the PI3K/Akt signaling pathway [130, 131].

### Gastric cancer (GC)

Gastric carcinoma is a prevalent malignancy worldwide and ranks as the fourth leading cause of cancer-associated deaths [132–134]. The occurrence of gastric cancer exhibits geographical variations worldwide, being most prevalent in Eastern Asia (specifically Japan and Mongolia) and Eastern Europe. Conversely, the incidence rates in Northern Europe and Northern America tend to be relatively low, comparable to those observed in African regions [135]. The expression of ALKBH5 and METTL3 was upregulated, whereas that of METTL14 was downregulated in GC [23, 35, 136, 137]. Patients with higher METTL14 expression had better overall survival (OS) outcomes [137]. The knockdown of METTL14 increased the proliferation, migration, and invasion of GC cells. METTL3-mediated m6A modification upregulated the expression of the THAP7-AS1 gene in GC cells. This upregulation was significantly correlated with positive lymph node metastasis and poor overall prognosis [35]. The increased expression of THAP7-AS1 also promoted the growth, migration, and invasion of GC cells [35]. The expression of ALKBH5 (a molecule that recognizes m6A modifications) decreased the stability of TP53TG1 mRNA and subsequently downregulated its expression. The decreased expression of TP53TG1 was associated with advanced clinical features of GC, such as a larger tumor diameter, poorer tumor differentiation, higher TNM stage, and increased lymph node metastasis, which ultimately led to a poorer prognosis. TP53TG1 interacts with cancerous inhibitor of protein phosphatase 2 A (CIP2A) and enhances its ubiquitination, thereby inhibiting the PI3K/AKT pathway [136]. The overexpression of the THAP7-AS1 gene intensified the inhibitory effects of CUL4B on the transcription of miR-22-3p and miR-320a, leading to an increase in the expression of PIK3CA, PIK3CD, and AKT3 genes. This, in turn, promoted the progression of GC. Decreased RNA m6A methylation stimulates the oncogenic PI3K/Akt pathway, leading to the development of malignant characteristics in gastric cancer cells [23]. METTL14 plays a crucial role in controlling abnormal m6A modification in GC. It inhibits the progression and metastasis of GC cells by deactivating the PI3K/AKT/mTOR pathway and EMT. Clinical and bioinformatic analysis indicated a decrease in the expression of the METTL14 gene in GC [137].

### Colorectal cancer (CRC)

CRC is the fourth leading cause of cancer-related deaths and is responsible for 9.2% of global deaths [138, 139]. It has been reported that roughly 41% of cases of colorectal cancer present in the proximal colon, alongside approximate incidences of 22% for the distal colon and 28% for the rectum [140]. In the year 2018, the number of individuals newly diagnosed with CRC was in excess of 1.8 million. This accounted for approximately 10.2% of all cancer diagnoses made annually. Furthermore, there were 881,000 fatalities attributed to CRC, making up 9.2% of global cancer deaths [141]. The expression of the METTL3 gene increased, whereas the expression of the METTL14 gene decreased in CRC [142, 143]. Patients with CRC who have high expression of METTL3 experience a decreased OS compared to those having low expression [142]. Patients with low METTL14 expression in CRC showed poor OS [143]. The decreased expression of METTL14 was strongly associated with metastasis of cancer to lymph nodes and other body parts, as well as the TNM stage of cancer. In contrast, overexpression of METTL14 inhibited the metastasis of CRC cells [143]. Mechanistically, METTL3 regulates EphA2 and VEGFA through an m6A-related mechanism. Knockdown of METTL3 increased the stability of EphA2 and VEGFA mRNA through a pathway that involves m6A-IGF2BP2/3. METTL3 promotes vasculogenic mimicry in CRC cells by modifying EphA2/VEGFA through the PI3K/AKT and ERK1/2 signaling pathways, both in vitro and in vivo [142]. METTL14 is involved in the repression of the SRY-related high-mobility-group box 4 (SOX4) through m6A and YTHDF2. Furthermore, the suppression of METTL14 in CRC can enhance the SOX4-mediated EMT process and activate the PI3K/Akt signaling pathway [143].

### Gallbladder cancer (GBC)

Gallbladder carcinoma is a type of cholangiocarcinoma that develops from the inner lining of the gallbladder [144]. Pancreatic cancer represents approximately 1.3% of the total cancer incidence worldwide and accounts for 1.7% of all cancer-related deaths [145]. The annual prevalence of carcinoma of the gallbladder in Australia is estimated to fall within the range of 3–4 cases per 100,000 individuals [146]. It is the most common type of cancer that affects the biliary tract and has the poorest overall prognosis [147]. Patients with GBC who have decreased levels of deoxycholic acid (DCA) showed a lower OS. Further, univariate Cox regression analysis demonstrated a significant correlation between the survival of patients with GBC and important parameters, such as liver invasion, TNM stage, distant metastasis, and T-classification. DCA binds to METTL3 in the METTL3–METTL14–WTAP protein complex, impairing its function. This results in a decline in miR-92b-3p expression, thereby inhibiting tumor growth in GBC. Therefore, DCA shows a therapeutic effect in GBC by decreasing the expression of miR-92b-3p and subsequently increasing the expression of PTEN [143].

### Hepatocellular carcinoma (HCC)

**Fig. 3:**
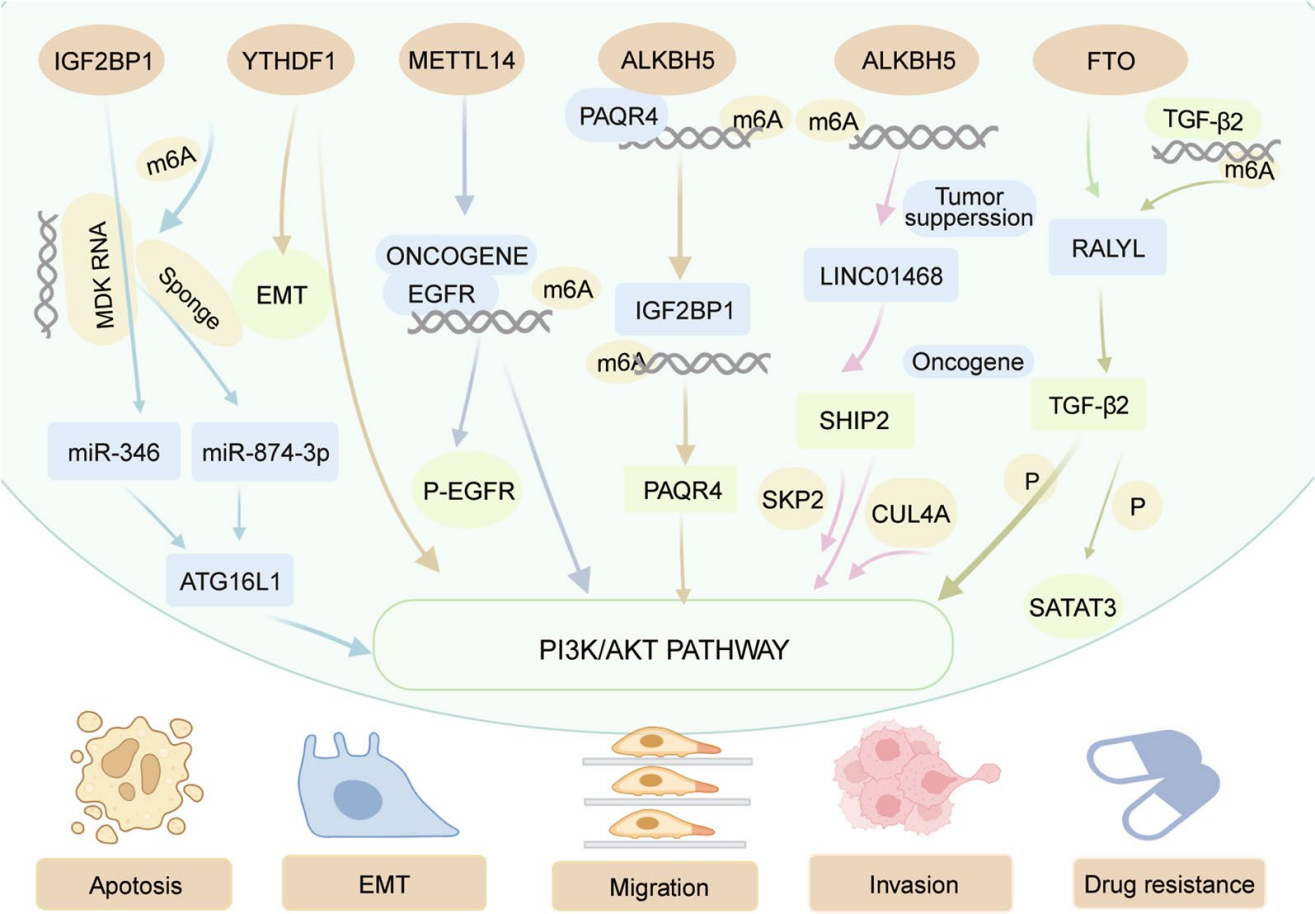
Mechanism of hepatocellular cancer progression involving m6A regulator and the PI3K/AKT pathway. FTO, IGF2BP1, YTHDF1, METTL14, and ALKBH5 are involved in promoting the proliferation, migration, and invasion of hepatocellular carcinoma cells by activating or inhibiting PI3K/AKT signaling pathways. Image created with BioRender (https://biorender.com/)

HCC is the third leading cause of cancer-related deaths globally, and liver cirrhosis is its primary predisposing factor [148, 149]. The collective contribution of Asian nations to the prevalence and fatalities related to liver cancer in 2020 accounts for 72.5% and 73.3% respectively, on a global scale. Over the given period between 2018 and 2020, the manifestation of liver cancer incidence and mortality within the Asian populace has exhibited variable patterns [150]. M6A modifications involved in the PI3K/AKT signaling pathway are significantly correlated with the initiation and progression of HCC [34, 151–154] (Table 2). The gene expression of FTO, IGF2BP1, and YTHDF1 is significantly increased, whereas the expression of METTL14 and ALKBH5 is decreased in HCC. Patients with HCC who have higher levels of YTHDF1 exhibited poorer overall survival compared to those with lower levels [34]. IGF2BP1 can bind to circMDK in vitro, and m6A modification plays a crucial role in enhancing the stability of circMDK mRNA. The expression of the MDK protein was elevated in HCC tissues, which correlated with a decreased 5-year survival rate [155]. The presence of RALY RNA binding protein like (RALYL) was significantly correlated with poor overall and disease-free survival outcomes [154]. The OS rates decreased in patients with high LINC01468 expression [153]. Patients with downregulated METTL14 in HCC showed a statistically significant poorer relapse-free, progression-free, and disease-specific survival [152]. ALKBH5 demethylates PAQR4, which promotes HCC progression through the PI3K/AKT signaling pathway [153]. The suppression of YTHDF1 expression decreased the invasiveness of HCC [34]. CircMDK is a tumor-promoting circRNA that enhances cell growth and suppresses apoptosis in HCC cells [155]. RALYL promotes cell motility and metastasis by inducing EMT [154]. The suppression of LINC01468 effectively impairs the chemoresistance and tumorigenesis of HCC [153]. Decreased concentration of the ALKBH5 protein effectively impaired the proliferative and invasive potential of HCC cells [151, 152]. Mechanistically, YTHDF1 promoted the progression of HCC by activating the PI3K/AKT/mTOR signaling pathway [34]. RALYL could control HCC stemness through STAT3-dependent upregulation of TGF-β2 [154]. The METTL14 protein inhibited the migration, invasion, and EMT of HCC cells by regulating the EGFR/PI3K/AKT signaling pathway in an m6A-dependent manner, and its downregulation promoted malignancy in HCC cells [152] (Fig. 3).

## Reproductive system neoplasms

### Ovarian cancer (OC)

Ovarian cancer currently holds the seventh rank among malignant tumors and the eighth position as a leading cause of cancer-related deaths in women worldwide [156]. The incidence of this malignancy exhibits significant variation with regard to geographical regions and population demographics. Notably, in 2012, the North European and American regions registered the highest prevalence of ovarian cancer, while Japan reported the least incidence [157]. Ovarian carcinomas include multiple neoplasms that are classified based their type and level of differentiation [158]. The expression of METTL3 was upregulated in OC [159, 160]. The elevated levels of RHPN1-AS1 are strongly correlated with the occurrence of distant metastasis and mortality in patients with OC. Patients showing high expression of RHPN1-AS1 had poor overall and disease-free survival rates [160]. The METTL3-induced m6A alteration of RHPN1-AS1 increased the stability of RHPN1‐AS1 in OC cells resistant to cisplatin [159]. RHPN1-AS1 is a cancerous long non-coding RNA in epithelial OC, and it promotes the proliferation, migration, and invasion of epithelial OC cells [160].

### Cervical cancer

Cervical cancer is the most prevalent cancer among women in developing nations, accounting for approximately a quarter of all cancers affecting females [161]. Globally, the prevalence and fatality rates associated with cervical malignancies are second only to those of breast cancer [162]. The expression of the METTL14 gene is decreased in cervical cancer [163]. The upregulation of METTL14 mRNA expression is significantly associated with improved OS in patients with cervical cancer. Downregulation of METTL14 inhibits the migratory and invasive capabilities of cervical cancer cells in vitro. The PI3K/Akt/mTOR signaling pathway is crucial in cervical cancer, including tumorigenesis and development [163].

## Urinary system neoplasms

### Renal cell cancer (RCC)

Renal cell carcinoma refers to a cluster of cancerous growths that originate from the epithelial cells of the renal tubules [164]. RCC represents approximately 90% of all malignant neoplasms arising from the kidney [165]. Around 20–30% of patients suffering from RCC are diagnosed at the metastatic phase, with an additional 20% experiencing relapse following their primary medical intervention [166]. The expression of METTL14 was notably decreased in clear cell renal cell carcinoma (ccRCC) tissues [167]. In contrast, the expression of IGF2BPs was significantly elevated in RCC tissues [168]. The downregulation of METTL14 was correlated with an unfavorable prognosis among patients diagnosed with ccRCC [167]. In contrast, the upregulation of WTAP and IGF2BPs showed a positive correlation with unfavorable prognostic outcomes in patients with RCC [168]. Overall, METTL14 exerts an inhibitory effect on the proliferation and migration of ccRCC cells in vitro. In contrast, WTAP and IGF2BPs promoted RCC metastasis [167, 168]. WTAP enhances the malignant progression of RCC by modulating the expression of S1PR3 through the activation of the PI3K/AKT pathway [168]. The upregulation of METTL14 reduced the phosphorylation levels of PI3K, AKT, and mTOR in both the Caki-1 and Caki-2 cell lines. These findings suggest that the overexpression of METTL14 inhibits the PI3K/AKT signaling pathway [167].

### Bladder cancer

Bladder cancer is a common malignancy in the urinary system and is one of the most prevalent types of cancer worldwide [169]. In the year of 2020, a total of 573,278 individuals were diagnosed with BLCA, culminating in 212,536 fatalities that were directly attributed to the disease [170]. The expression of YTHDC1 was reduced in patients with bladder cancer who had undergone chemotherapy. Moreover, the low expression of YTHDC1 suggested poor sensitivity to cisplatin and was associated with worse overall survival in patients with bladder cancer. This observation was further corroborated by the consistent reduction in PTEN levels and upregulation of p-AKT after cisplatin treatment. Notably, silencing YTHDC1 in bladder cancer cells decreased the expression of PTEN and activated the PI3K/AKT pathway [171].

### Prostate cancer (PCa)

PCa is the most common noncutaneous malignancy among males worldwide [172]. The incidence of prostate cancer varies across geographical regions and ethnicities globally. Notably, Black males possess the highest reported incidence rates of prostate cancer worldwide [173, 174]. The expressions of the METTL3 and YTHDF1 genes were significantly upregulated in PCa [175, 176]. Patients with PCa who have high expression of METTL3 exhibited higher rates of tumor recurrence compared to those with low expression of METTL3. However, OS rates between these two groups of patients were not significantly different [175]. Conversely, a notable association was observed between increased expression of YTHDF1 and unfavorable OS rates [176]. The suppression of METTL3 impaired cellular activities associated with cell proliferation. Conversely, overexpression of METTL3 stimulated the growth and invasion of PCa cells. The overexpression of YTHDF1 facilitated the initiation and dissemination of PCa, leading to increased tumor formation and metastasis [175]. PCa tumorigenesis was facilitated by the activation of the PI3K/AKT signaling pathway through the METTL3-mediated m6A modification of the lncRNA MALAT1 [175]. The translational efficiency of PLK1 in prostate cancer is controlled by YTHDF1, which is activated by ELK1. This regulation is dependent on m6A and affects the activation of the PI3K/AKT signaling pathway [176].

### Nervous system neoplasms

#### Glioblastoma

The prevalence of primary malignancies in the brain stands at around 7 cases per 100,000 individuals, with glioblastomas accounting for approximately 49% of these cases. The majority of patients succumb to this condition as it progresses [177]. Glioblastomas are the most common type of malignant primary brain tumors and have a significant impact on morbidity and mortality [178]. The abundance of m6A-modified lncRNA WEE2-AS1 increased in glioblastoma multiforme. In vivo experiments revealed that the downregulation of WEE2-AS1 significantly inhibited tumor growth. METTL3-induced m6A modification increased the stability of WEE2-AS1 in the presence of IGF2BP3. Therefore, the expression of IGF2BP3 affects the expression and stability of WEE2-AS1. The lncRNA WEE2-AS1 can act as a scaffold for RPN2, and the resulting WEE2-AS1/RPN2 complex activates the PI3K-AKT signaling pathways to promote the progression of glioblastoma [179].

### Retinoblastoma (RB)

Retinoblastoma is a frequently occurring tumor that affects the eye in childhood. If left untreated, it can have a fatal outcome [180, 181]. The global incidence of retinoblastoma is estimated to be 1 in every 16,000 to 20,000 live births [182]. The majority of instances are identified prior to reaching the age of five and constitute a 3% proportion of malignant neoplasms diagnosed during childhood [183]. The mRNA and protein expression of METTL3 is upregulated in RB. The migratory and invasive characteristics were significantly attenuated in RB cells with down-regulated METTL3. Notably, the upregulation of METTL3 induced by rapamycin had favorable impacts on RB cells by suppressing the PI3K/AKT/mTOR signaling pathway. Therefore, the METTL3 enzyme plays a regulatory role in the proliferation, apoptosis, migration, and invasion of RB cells by modulating the PI3K/AKT/mTOR signaling pathway [184].

## Respiratory system

### Nasopharyngeal carcinoma (NPC)

Nasopharyngeal carcinoma originates from the cells that line the upper part of the throat and the back of the nose. It typically affects the head and neck region [185]. Based on the findings of the International Agency for Research on Cancer, it was reported that a total of 129,079 novel incidences of nasopharyngeal carcinoma were documented in 2018 [186]. Nasopharyngeal carcinoma exhibits a notable spatial distribution pattern and is highly predominant in regions of eastern and southeastern Asia [187]. The expressions of METTL3 and YTHDC2 were upregulated in NPC cells [188, 189]. A statistically significant correlation was observed between the diminished expression of YTHDC2 mRNA and the favorable prognosis of NPC [189]. Patients with elevated ZFAS1 expression demonstrated poor overall and disease-free survival outcomes [188]. ZFAS1 promotes the proliferation and metastasis of NPC cells in vitro and in vivo. This effect is achieved by regulating autophagy levels through modulation of the miR-100-3p/ATG10 pathway [188]. Specifically, ZFAS1 acts as a sponge for miR-100-3p, which, in turn, increases the expression of ATG10. Additionally, ZFAS1 modulates the PI3K/Akt/mTOR pathway to influence the level of autophagy [188]. The reduction of YTHDC2 decreases the protein concentration of IGF1R and inhibits downstream PI3K-AKT/S6 signaling. Conversely, the upregulation of YTHDC2 increased the level of the IGF1R protein and activated the PI3K-AKT/S6 signaling pathway [189].

### Lung adenocarcinoma (LUAD)

Lung cancer is the second most frequently diagnosed malignancy and the leading cause of cancer-related fatalities in the United States [190]. Lung cancer remains one of the most commonly diagnosed malignancies globally, representing the primary cause of cancer-related mortality. It is estimated that every year, there are approximately 2 million new cases of lung cancer, leading to 1.76 million deaths [191]. The expression of the METTL3 gene is reduced, whereas that of the IGF2BP2 gene is increased in lung cancer [192, 193]. Increased levels of IGF2BP2 are correlated with an unfavorable prognosis in LUAD. In contrast, repression of IGF2BP2 expression alleviates the growth, migration, invasion, and angiogenesis of LUAD [193]. The inhibition of METTL3 decreases the proliferation, migration, and invasion of LUAD cells. The depletion of METTL3 induced apoptosis in LUAD cells by regulating the expression of apoptosis-associated proteins [192]. LUAD cell-secreted exosomes facilitate the transfer of IGF2BP2 to neighboring endothelial cells, promoting the initiation of the PI3K-AKT signaling pathway, which is crucial for angiogenesis [193].

## Motor system tumor

### Osteosarcoma

Osteosarcoma is the most prevalent primary bone sarcoma, which mainly affects children, adolescents, and young adults. It also commonly occurs among elderly individuals [194]. The expression of WTAP was significantly elevated in osteosarcoma tissue. This upregulation was closely associated with the clinicopathological features of patients with osteosarcoma and was also a strong predictor of a poor prognosis in this patient population. Increased expression of WTAP was significantly correlated with larger tumor size, an increased incidence of metastasis, and a higher TNM stage, thereby indicating a poor prognosis. The HMBOX1 is involved in the proliferation and metastasis of osteosarcoma, which is mediated by WTAP in vitro. WTAP and HMBOX1 modulate the PI3K/AKT pathway to regulate the growth and metastasis of osteosarcoma [195].

## Therapeutic implications of the association between m6A regulators and the PI3K/AKT signaling pathway

**Fig. 4:**
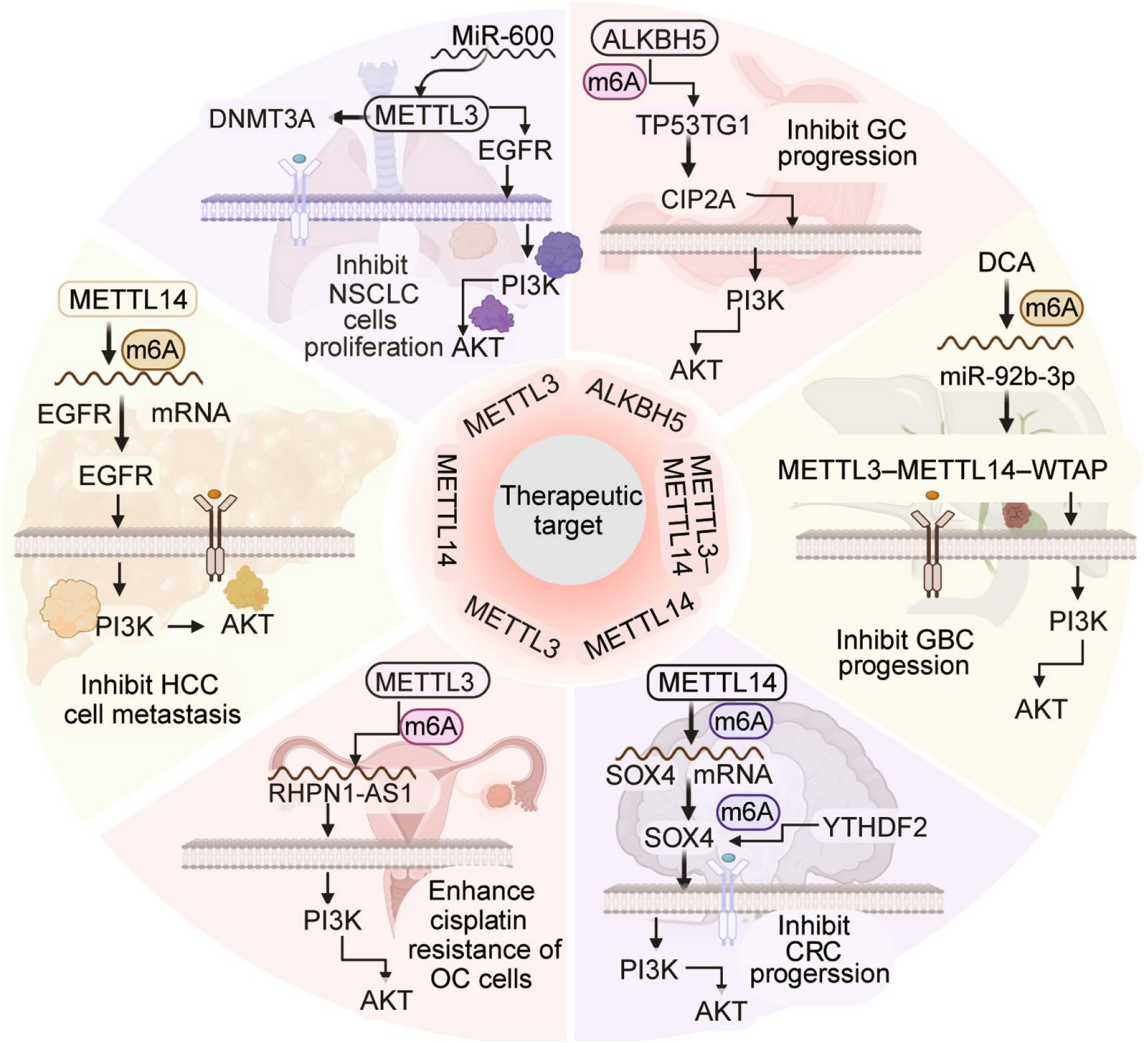
Therapeutic implications of the association between m6A regulators and the PI3K/AKT signalling pathway. The METTL3, METTL14, and ALKBH5 proteins, which are regulators of the m6A modification in RNA molecules, have been shown to have the ability to modulate the activity of the PI3K/AKT signaling pathway in different types of cancer. This regulation results in a consequential impact on the progression, metastasis, and proliferation of the cancer cells, thus indicating a desirable avenue for therapeutic optimization. Image created with BioRender (https://biorender.com/)

Given the involvement of the PI3K/AKT signaling pathway in conferring resistance to chemotherapy and radiotherapy, several researchers have focused on targeting this pathway for cancer treatment [64]. Moreover, several authors have linked m6A modification to chemotherapy and radiotherapy resistance [196]. M6A regulators and the PI3K/AKT pathway interact with each other in cancer, and targeting this interaction can be a promising strategy for overcoming treatment resistance [197] (Fig. 4). The inhibition of METTL3 decreased aggressive tumor phenotypes of PDAC, potentially through the attenuation of m6A modification on the DDX23 mRNA. Therefore, targeting the METTL3/DDX23 pathway can reverse GEM resistance in PDAC [130]. The protein TP53TG1 interacts with CIP2A, resulting in its degradation through ubiquitination and ultimately inhibiting the PI3K/AKT pathway. TP53TG1 is crucial in suppressing the progression of GC and thus represents a key therapeutic target for GC treatment [136]. DCA may act as a tumor suppressor in GBC by inhibiting the maturation of miR-92b-3p. This suggests that DCA treatment could potentially offer a novel therapeutic approach for GBC [198]. An intervention focused on METTL3 and IGF2BP2/3 may present a promising diagnostic or prognostic target for vasculogenic mimicry-targeting medications in colorectal cancer treatment [142]. The reduction of METTL14 contributes to the promotion of tumor metastasis in CRC. Therefore, METTL14 can be a valuable prognostic biomarker and an effective therapeutic target for CRC treatment [143]. The expression of METTL14 was markedly reduced in HCC and showed a significant correlation with cancer prognosis. Additionally, METTL14 was observed to inhibit the migration, invasion, and EMT of HCC cells by modulating the EGFR/PI3K/AKT signaling pathway in an m6A-dependent manner. Therefore, targeting the METTL14/EGFR/PI3K/AKT signaling pathway may represent a promising therapeutic approach for preventing HCC metastasis [152]. The m6A modification induced by the ALKBH5 enzyme stabilizes and increases the levels of LINC01468 mRNA. Further, LINC01468 promotes lipogenesis, leading to the progression of HCC by facilitating the degradation of SHIP2 through its interaction with CUL4A. This highlights a potential therapeutic approach for HCC by targeting the LINC01468/SHIP2 axis [153].

A pathway involving miR-600 and METTL3 is involved in the progression of non-small cell lung cancer (NSCLC), wherein miR-600 inhibits the expression of METTL3, thereby counteracting its positive effect on NSCLC progression. Specifically, miR-600 achieves this effect by downregulating the expression of METTL3, thereby inhibiting lung cancer. These results suggest the possibility of targeting METTL3 as a novel therapeutic approach for treating lung cancer [192]. The YTHDC2 protein interacts with the IGF1R mRNA and enhances the initiation of mRNA translation, which, in turn, activates the IGF1R/AKT/S6 signaling pathway. YTHDC2 is involved in enhancing cellular resistance to radiotherapy by activating the IGF1R/AKT/S6 signaling pathway. Therefore, it may be a promising target for therapeutic interventions aimed at sensitizing cancer cells to radiation [189]. The WEE2-AS1 gene enhances the stability of the RPN2 protein by inhibiting the CUL2-mediated ubiquitination of RPN2 at K322. This initiates the PI3K/AKT signaling pathway, thereby promoting the glioblastoma progression. The suppression of this signaling cascade by inhibiting WEE2-AS1 enhanced the potency of dasatinib, suggesting a promising strategy for optimizing targeted combination therapy [179].

## The impact of m6A modification on the regulation of various signaling pathways in cancer

**Fig. 5:**
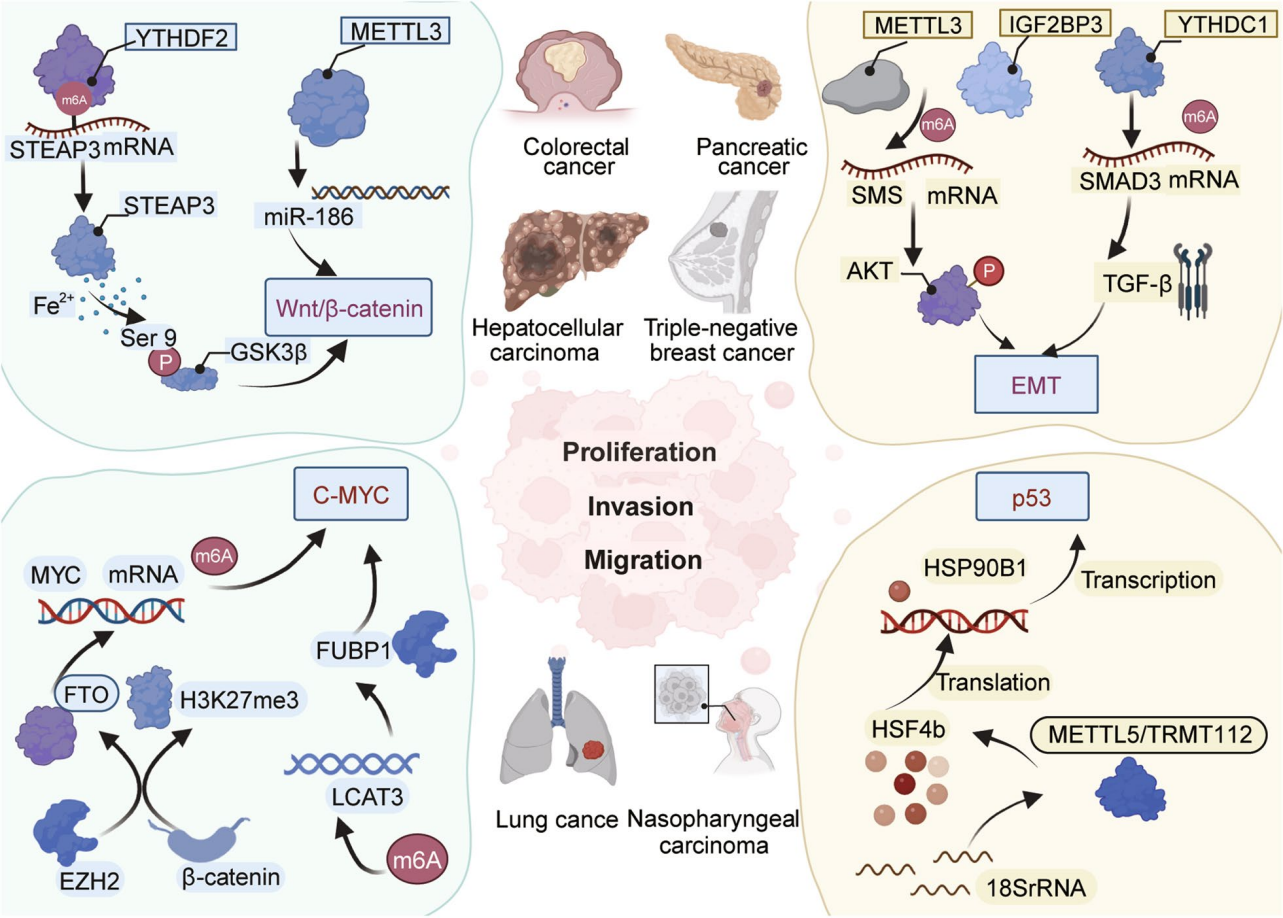
The m6A modification have been implicated in several other canonical pathways, such as C-MYC, Wnt/β-catenin, p53, and the EMT. The METTL3, YTHDF2, IGF2BP3, FTO, and other regulators of m6A modification have the ability to influence various pathways such as p53, C-YMC, Wntβ-catenin, and EMT. They engage in diverse mechanisms to participate in the regulation of tumor proliferation, migration, and invasion in colorectal cancer, lung cancer, pancreatic cancer, breast cancer, and other types of cancer. Consequently, these regulators exert substantial influence on the initiation and progression of tumors. Image created with BioRender (https://biorender.com/)

The m6A modification have been implicated in several other canonical pathways, such as C-MYC, Wnt/β-catenin, p53, and the EMT (Fig. 5). The Wnt/β-catenin pathway consists of a group of proteins that have significant functions in the development of embryos and the maintenance of adult tissue balance. The disturbance of Wnt/β-catenin signaling frequently results in different types of cancer [199, 200]. According to previous research, a long lncRNA known as STEAP3-AS1, induced by hypoxia, was responsible for promoting the expression of STEAP3 by interacting with the protein YTHDF2, thereby preventing m6A-mediated degradation of STEAP3 mRNA. The elevated expression of STEAP3 leads to an increase in cellular Fe2 + concentration, which initiates the Ser 9 phosphorylation of GSK3β. This activation of the Wnt/β-catenin signaling pathway results in the acceleration of CRC progression [201]. Li and colleagues noted a noteworthy decrease in the expression of circFBXW7 in cell lines that had developed resistance to Osimertinib. The circFBXW7 molecule exerted a potent inhibitory effect on the stem cell properties of LUAD and also countered resistance to TKIs by regulating the Wnt signaling pathway. This biological action was attributed to the circFBXW7-185AA fragment, which facilitated the ubiquitination and inhibition of β-catenin [202]. Previous research has demonstrated that METTL3 is upregulated in both hepatoblastoma (HB) tissues and cell lines. This gene has been functionally characterized as an oncogene in HB. Additionally, miR-186 has been identified as a direct target of METTL3, with ectopic overexpression of miR-186 leading to a significant decrease in aggressive tumor phenotypes in HB. The miR-186/METTL3 axis is crucial for the initiation and progression of HB through the regulation of the Wnt/β-catenin signaling pathway [203].

The c-myc proto-oncogene comprises a crucial element of the cell’s proliferative apparatus, and its uncontrolled expression is associated with the majority of neoplasms [204–206]. A previous study has indicated that the upregulation of LCAT3 was caused by m6A modification, which was mediated by METTL3 and consequently resulted in the stabilization of LCAT3. The knockdown of LCAT3 led to cell cycle arrest in the G1 phase. Mechanistically speaking, LCAT3 facilitated the recruitment of Far Upstream Element Binding Protein 1 to the MYC far-upstream element sequence, thereby triggering MYC gene transcription, which promoted the proliferation, survival, invasion and metastasis of lung cancer cells [207]. A previous study has shown that the expression of FTO is reduced in lung adenocarcinoma. When FTO expression is downregulated, it significantly increases the levels of m6A modification in the mRNAs of numerous genes involved in important pathways, especially those related to metabolism, such as MYC. The increased m6A modification on MYC mRNA leads to the recruitment of YTHDF1 binding, which in turn promotes the translation of MYC mRNA. This results in enhanced glycolysis and proliferation of tumor cells, ultimately contributing to tumorigenesis [208].

The p53 protein is a transcription factor that functions to safeguard cells against cellular stress, primarily by regulating the expression of genes that promote cell cycle arrest, DNA repair, programmed cell death, cellular senescence, or altered metabolism [209]. The activation of p53 presents a potential therapeutic strategy for the manipulation of disease pathologies [210–212]. In a prior investigation, it was demonstrated that the modification of m6A1832 at the 18S rRNA by METTL5/TRMT112 selectively regulates the translation of mRNAs containing 5′ terminal oligopyrimidine motifs by promoting the assembly of 80S ribosomes through the facilitation of RPL24-18S rRNA interaction. Moreover, METTL5 enhances the translation of HSF4b, which in turn activates the transcription of HSP90B1. The formed HSP90B1 protein binds to the gain-of-function p53R280T protein, thereby preventing its degradation through ubiquitination. As a result, the tumorigenesis and chemoresistance of nasopharyngeal carcinoma are promoted [213].

The process of transitioning from epithelial to mesenchymal phenotype has become recognized as a crucial factor in determining tumor cell invasion and metastasis [214, 215]. This malleable process involves the initial acquisition of invasive capabilities by epithelial cells, allowing for migration into the bloodstream through the transformation into mesenchymal cells, also known as EMT [216, 217]. Tan and colleagues discovered that the protein YTHDC1 has an important role in promoting the spread of TNBC. YTHDC1 achieves this by facilitating the export of SMAD3 from the cell nucleus and increasing its expression, which in turn leads to activation of the TGF-β signaling pathway. Additionally, YTHDC1 is crucial for TNBC progression, as it helps protect cancerous cells and promotes the transition to a more aggressive form of cancer known as EMT through SMAD3 [218]. Spermine synthase (SMS) is an enzyme involved in the production of polyamines [219, 220]. In the context of pancreatic cancer, the expression of SMS is increased. It has been discovered that both METTL3 and IGF2BP3 directly target SMS and bind to its m6A modification sites, preventing mRNA degradation. Excessive SMS activity hampers the build-up of spermidine by converting it to spermine. This, in turn, triggers the phosphorylation of serine/AKT and activates the EMT signaling pathway, leading to the inhibition of pancreatic cancer cell proliferation and invasion [221].

## Conclusion

M6A modification of RNA can regulate the activity of genes involved in the PI3K/AKT signaling pathway. Dysregulation of m6A modification can contribute to the aberrant activation of the PI3K/AKT pathway and the development and progression of cancer. Cancer is a complex disease that is governed by multiple regulatory pathways and involves numerous molecular regulators. Among these molecular regulators are m6A regulatory proteins, which have been implicated in various other regulated pathways such as C-MYC, Wntβ-catenin, p53, and EMT. Through their involvement in these pathways, m6A regulatory proteins are capable of exerting profound effects on the fundamental hallmarks of cancer, including proliferation, invasion, and metastasis. In addition, m6A modification can also regulate the expression of several other genes related to cancer. For example, m6A modification of the mRNA encoding MYC, a key oncogene, enhances its translation and subsequent protein expression, leading to increased migration and invasion of cancer cells. Therefore, targeting m6A modification can have substantial therapeutic potential in cancer treatment. However, further research is needed to understand the molecular mechanisms underlying the impact of m6A modification on the PI3K/AKT signaling pathway and its potential as a pan-cancer therapeutic target.

## Data Availability

Not applicable.
